# Genetic variation in hydrogen cyanide potential of perennial sorghum evaluated by colorimetry

**DOI:** 10.1002/pld3.448

**Published:** 2022-10-21

**Authors:** Shakirah Nakasagga, Seth C. Murray, William L. Rooney, Catherine Barr, Pheona Nabukalu, Stan Cox, Leo Hoffmann

**Affiliations:** ^1^ Department of Soil and Crop Sciences Texas A&M University College Station Texas USA; ^2^ Texas A&M Veterinary Medical Diagnostic Laboratory College Station Texas USA; ^3^ The Land Institute Salina Kansas USA; ^4^ Department of Horticulture Sciences University of Florida Gainesville Florida USA

**Keywords:** colorimetry, hydrogen cyanide potential, perennial sorghum, visual assessment

## Abstract

Both annual and perennial sorghum biomass serve as important forage for ruminant animals around the world. Unfortunately, sorghum can produce hydrogen cyanide (HCN), which, if occurring in high enough concentrations, can be toxic or lethal to animals that consume it. The objectives of this study were to develop a fast and inexpensive colorimetric assay to measure the hydrogen cyanide potential (HCN‐P) as well as to compare this with existing visual assays while assessing the range of variation for HCN‐P among perennial and annual sorghum biomass. The HCN‐P of 100 sorghum lines derived from an interspecific hybridization program was determined over 2 years (establishment and regrowth) using both visual and colorimetric assays. Visual assessment underestimated the HCN‐P and was less accurate than colorimetry. Repeatability for HCN‐P across all sampling dates was functionally zero in the visual assessment and low for the colorimetric assay. This was mostly explained by the significant pedigree × year interaction effects and growth stage. Growth stage substantially influenced HCN‐P, which should be considered when feeding animals on fresh forage.

## 1 INTRODUCTION

Several sorghum species are used as forage in animal production systems worldwide. Specific cultivars and hybrids of *Sorghum bicolor* are planted annually for grazing and baling. Among the perennial sorghum species, *Sorghum halepense* (Johnson grass) was originally introduced to the United States as a forage species (McWhorter, 1971) and two other perennial sorghum species (*Sorghum propinquum* and *Sorghum almum*) have potential as a perennial forage (De Wet, 1978; Doggett, 1976; Pritchard, 1965).

Perennial sorghums are characterized by a deep rooting system with rhizomes for temporal storage of carbohydrates, which is used as an energy source for plant regeneration (Cox et al., 2018). Rhizomes are reported as the primary factor associated with overwintering in perennial sorghum and able to reduce costs associated with annual crop establishment (Sanderson & Adler, 2008). The perennial rooting system combined with the clumping nature of growth can reduce soil erosion and subsequently increase soil organic carbon (Nabukalu & Cox, 2016), but it is also noteworthy that rhizomes are a contributing factor to the aggressive behavior of perennial sorghum. As such, Johnsongrass (*S. halepense*) has colonized large land masses and become one of the 10 worst weeds of field crops (Alex et al., 1979). However, progenies of annual × perennial crosses have been reported to have less than 10% rhizome mass, sufficient for overwintering, but not enough to become aggressive weeds (Piper & Kulakow, 1994).

Most sorghum accessions produce the cyanogenic glucoside dhurrin in the aboveground biomass. When stress occurs (caused by abiotic and/or biotic factors), enzymatic reactions cleave the dhurrin molecule to produce hydrogen cyanide (HCN). This HCN production is thought to have evolved as a defense against insect herbivory (Etuk et al., 2012). Although dhurrin does provide some protection, it can be toxic to ruminants in high concentrations (Krothapalli et al., 2013). Further, some unselected perennial sorghum types accumulate more dhurrin than annual grain and forage types (Rhykerd & Johnson, 2007).

Variation has been found for levels of HCN‐P in domesticated *S. bicolor* and wild *S. halepense* (Hayes et al., 2016; Nicollier et al., 1983) but has not been investigated for progenies of *S. bicolor* × *S. halepense*. To select for lower HCN‐P in these materials, cost‐effective methods are needed for measurement. Various protocols have been used to estimate HCN‐P in crop biomass (Table 1). The Feigl–Anger and picrate paper methods are inexpensive, subjective, and qualitative approaches to rapidly screen large numbers of samples through visual assessment of color intensity associated with increasing HCN concentrations (O'Brien et al., 1994). Biochemical and spectrometric methods provide quantitative measures although they are expensive and time consuming, which makes it difficult to screen large populations (Akazawa et al., 1960; Fox et al., 2012; Reddy et al., 2016). Ultimately, accurate quantitative measures are important for data manipulation and statistical analysis, but price and rapid testing are necessary for high‐throughput screening (Punch, 2013; Walter & Andersen, 2013). The objectives of this study were to (1) evaluate if colorimetry could be used to make visual HCN assays more quantitative and repeatable and (2) assess the range of variation for HCN‐P among breeding lines of perennial sorghum.

**TABLE 1:** Methods used for estimating HCN‐P in different crop species

Type of method	Quantitative		
	Cost of equipment ($)	Time/sample	Species
Spectrometry Absorbance spectrophotometer microplate reader and near‐infrared spectrometer (Fox et al., 2012)	>2600	Microplate reader (37°C for 15 h)	Forage sorghum (*Sorghum bicolor*)
		NIR scan (1–2 min)	
Picrate paper with spectrometry (Burns et al., 2012)	>2800	Absorbance (30°C for <12 h)	Cassava (*Manihot esculenta*)
Biochemical methods (Akazawa et al., 1960)	900	16–24 h	*Sorghum vulgare* (*Sorghum bicolor*)
	Semiquantitative		
Feigl and Anger densitometry (Reddy et al., 2016)	715	1.30 h	Grain sorghum (*Sorghum bicolor*)
	Qualitative		
Indicator paper test			
Feigl and Anger test	425	20–35°C for 3–12 h	Cassava
Picrate paper test (Buck et al., 1973; O'Brien et al., 1994)	205	20–35°C for 10–12 h	(*M. esculenta*)

## 2 MATERIALS AND METHODS

### 2.1 Germplasm

This study evaluated 100 sorghum pedigrees (genotypes), primarily comprising 79 BC_1_F_1_ progenies of BTx623/Gypsum‐ an interspecies cross of tetraploid *S. bicolor* (BTx623) and *S. halepense* (gypsum). These progenies were selected in Salina, Kansas, for both perenniality and grain productivity using methods described by Nabukalu a Cox (2016). In addition, 14 progenies derived from another interspecific cross between BTx623/*S. propinquum* (Washburn et al., 2013), pure lines of *S. halepense* (1) and (2) *S. bicolor* (1‐BTx623 diploid, 1‐BTx623 tetraploid line), and four hybrid grain sorghums (RTx436, RTx437, NP32_PI535776, Low‐HCN‐PRP2B) (Rooney et al., 2003) were also included (Table 2).

**TABLE 2:** Germplasm used for the study including the different species and interspecific combinations

*Sorghum bicolor*	*S* *orghum halepense*	*S. bicolor/S. halepense*					*S. bicolor/S. propinquum*
RTx437	GYPSUM‐9	S1662 > R126B	S2172‐5‐R506	S1662 > 153H‐R54	S2126 > R147B	S1776 > R349B	S3199‐B1‐PR135A
RTx436		S14PR‐020C	S1820‐1‐614	S1327 > 230B	S1814 > R452B	X1206 > R477	S3195‐A3‐PR14
BTX623(4X)		S1662 > R153G	S2371 > R519‐R124	X814 > R003	S1852 > 015E	S1776‐R331	S3182‐B7‐PR178A
LowHCN		S1662 > R040A‐R161	X1083‐021	S2163‐1‐065‐R190	S2372‐4‐R650A	S1852 > 015B‐R171B	S3167‐B1‐PR10G
NP32‐P15355776		S2372‐5‐R651	S1662 > 246A	S1327 > R144	S1477 > R175A	S1477 > 605A	X814‐201A‐PR101C
BTX623		S2002 > 269D	X814 > R209	S1662 > R512A‐R98	X999 > R305	S1344 > R036	S3182‐B3‐PR39B
		S2172‐7‐R475DW	S1843‐8‐578C	X1206‐054	X814‐201C‐R17	X754‐198	S1465 > 587A‐PR125A
		S1776 > 349C	S2607‐1‐R64A	X38‐18‐R4193	X814‐201B‐R523	S2371–81‐R606A	CS14‐PSOR‐091‐03
		S1661 > 089	S1346 > 174A	S1438 > 070A	S1465 > R120D	S1776 > R174*	S3317‐B2‐PR65A
		S1327 > 159A	S1477 > R216	X999 > 177B	S1662 > 216	X999 > R485	S3322‐C6‐PR67
		S1479 > R771A	X1210‐354	S2172‐5 > R312	S2097‐4‐137	S1662 > R554B	CS14‐PSOR‐080‐15
		X1092‐154	X799 > 105	S1438 > R302	S14PR > R181	S2371‐98‐R623	CS14‐PSOR‐022‐15
		S20693 > BK126	S1341 > R315	S1479 > R334B	X999R348B	S1662 > R040A	X814‐201B‐PR9C
		S1479 > R334B‐R45	S2171‐23‐R545A	S1312 > R97A	S1383 > 046F	S1646 > R46	S3188‐B6‐PR46A
		S2092‐1‐115BK‐R86	S1852 > 015B‐R171A	S2125 > 245B	S2163 > R186B‐R129G	S1776 > R65	
		X999 > R393	S2371‐23‐R545C	S2172 > R187	S1662 > R246C		

### 2.2 Experimental design and establishment

The pedigrees were planted at the Texas A&M AgriLife Research Farm in Burleson County, College Station, Texas. These trials were planted on April 2, 2019 (seed year), and maintained through 2020 (regrowth year). Annual sorghum (*S. bicolor*) was replanted on April 8, 2020. A randomized complete block design with two replications and single row plots of 6 m length and 1.5 m wide was used. Agronomic practices standard for forage sorghum production in Texas were followed (Schnell et al., 2019) throughout the growing season to maturity. The trials were harvested on a plot level using a John Deere 7300 forage harvester mid‐September and end of August in 2019 and 2020, respectively.

### 2.3 Hydrogen cyanide estimation

Because previous reports documented that HCN‐P varied based on growth stages and temperatures, HCN‐P was estimated at different growth stages and weather conditions with the goal of finding sorghum pedigrees low in HCN‐P at all times (Bahrani & Deghani, 2004; Barhnhart & Hartwing, 1993; Zahid et al., 2012). At each sampling, the biomass tested was leaves because they are known to accumulate dhurrin more than any other plant part (Gleadow et al., 2016). Younger leaves were selected, particularly the third leaf from the shoot (O'Donnell et al., 2013). One whole leaf was harvested from 10 different plants per plot (Loyd & Gray, 1970) and packaged into a ziplock bag and stored into a cooler from which fresh samples were tested for HCN‐P immediately in the laboratory. Fresh tissue was used because processes such as freeze‐drying samples are known to alter HCN‐P concentrations (Gleadow et al., 2012).

In the establishment year (2019), biomass sampling for HCN‐P occurred in late summer (September 3–6) when the plants were at physiological maturity stage (grains with a black layer at the bottom of the kernel were found in more than half of the plants within a plot). At this time, daily air temperatures ranged from 21 to 38°C, soil temperature ranged from 27 to 30°C, and relative humidity ranged from 28% to 92%. Further, the plots were under drought and heat stress.

In 2020, HCN‐P was first sampled in late spring May (27–29) when sorghum plants were at the five‐ to seven‐leaf stage (vegetative), daily air temperatures ranged from 18 to 30°C, soil temperature ranged from 26 to 27°C, and the relative humidity was 48%–99%. Third and fourth sample dates were collected July (1–3) at 50% flowering (stigma were found in more than half of the plants within a plot) and at physiological maturity (August 18–20) when daily air temperatures ranged from (28–29°C and 30–31°C), soil temperature (27–28°C and 31–32°C), and relative humidity (71%–76% and 65%–71%), respectively.

### 2.4 Visual assessment of HCN‐P

The visual color assessment of HCN‐P was based on the modified protocol of Buck et al. (1973). An independent HCN curve was built by mixing increasing amounts of amygdalin (synthetic cyanogenic glycoside originally found in bitter almonds) with a fixed amount of β‐glucosidase (enzyme) to visually assess color change using indicator paper. The indicator paper was prepared by dipping filter paper strips of uniform dimensions in picric acid reagent (aqueous 5% sodium carbonate with 0.5% picric acid) and blotting to dampness on paper towels. Aqueous solutions were prepared such that 1 ml solution would contain zero to 30 mg amygdalin and 1 ml of each solution was added to a different Erlenmeyer flask. An aqueous solution containing 1 mg/ml β‐glucosidase was prepared, and 100 μl was added to the opposite side of each flask containing the amygdalin solutions. An indicator paper was suspended in each flask, a stopper was firmly inserted, and the two solutions were mixed and incubated for 1 h at 37°C ± 2°C. Actual HCN concentration was computed from the amygdalin considering a ratio of 1 HCN: 1 amygdalin. HCN has a mass weight of 27.03, whereas amygdalin is 309.27 g.

**FIGURE 1:**
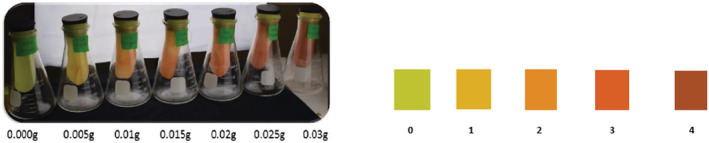
Visual assessment of indicator paper color change with varying amygdalin contents (left) and color scale (right).

The visual scale is based on the intensity of color change (Figure 1). An excess of amygdalin allowed the same amount of β‐glucosidase to produce a higher intensity red color, which upon computation gave the actual amount of HCN released following each enzymatic reaction.

For each plot, 300 g was weighed from the 10 whole leaves harvested per plot and cut into 1 cm pieces and placed into a 250‐ml Erlenmeyer flask. Chloroform (1.5 ml) was added to the sample flask to create a vapor to lyse cells and an indicator paper suspended by the tightly inserted stopper. A positive control was also prepared by adding 100 μl β‐glucosidase to 1000 μl of aqueous 1 mg/ml amygdalin. Samples were incubated in batches and scored. The color scale (Figure 1) was interpreted using a standard scale (Table 2).

### 2.5 Quantitative estimates of HCN‐P using colorimetry

A colorimetric procedure described by Sen et al. (2008) was modified for testing. Herein a colorimeter was used to get a quantified color change of HCN instead of a flatbed scanner that was used to quantify hydrogen sulfide in Sen et al. (2008). Test strips were exposed to a Minolta CR‐310 Chroma meter to measure the color change using the *L*, *a*, and *b* values obtained before and after incubation of the samples. The *L* value represents lightness of the surface with values ranging from 0 to 100, whereas a and b values are chromatic coordinates of the red‐green vector and blue‐yellow vector, respectively, both ranging from −120 to 120 (Papadakis et al., 2000). A quantified color change (ΔE) or the total difference was computed from these values by taking the difference between the post‐ and pre‐incubation images (Equation 1).

(1) $$\Delta E=\surd {\left(L_{2}-L_{1}\right)}^{2}+{\left(a_{2}-a_{1}\right)}^{2}+{\left(b_{2}-b_{1}\right)}^{2}$$

### 2.6 Data analysis

A HCN colorimetric curve was built based on the change in color (ΔE) compared with the actual HCN released from the different amygdalin concentrations. Correlations were run between HCN from the amygdalin and the values from the colorimetry. The values from the colorimetry were dependent on the standard as described earlier on in Section 2.4. The HCN concentration was derived from the amygdalin considering a ratio of 1 HCN: 1 amygdalin. HCN has a mass weight of 27.03, whereas amygdalin is 309.27 g. For example 0.005 g amygdalin = (.005/309.27)*27.03, giving 0.00044 g HCN. *L*, *a*, and *b* values were regressed against actual HCN to test the relationship in JMP® Pro Version 15 (SAS Institute Inc., Cary, NC, 2016). A frequency distribution for color across all plots was made to assess how many fell under each category on the color scale. Subsequently, comparisons were made with the colorimetric distribution.

The colorimetric measure and visual assessment assay were compared at the four data points (2019 physiological maturity and 2020 [vegetative (five‐ to seven‐leaf stage), flowering and physiological maturity]) using variance component analysis to estimate error variation and determine precision between the two methods. Correlation analysis was also run to assess the relationship between the colorimetric and visual assessment assay. Repeatability for HCN‐P was calculated as in Equation (2).

(2) $$\text{Repeatability}=\frac{\text{pedigree variance}}{\text{pedigree variance}+\left(\frac{\text{pedigree}\;x\;\text{year variance}}{\mathrm{no}.\text{ofyears}}\right)+(\text{error}/\left(\mathrm{no}.\text{of replicationx}\;\mathrm{no}.\text{of years}\right)}$$

A combined analysis across 2019 (physiological maturity, September 3–6) and 2020 (vegetative, May 27–29) for the quantified color change (ΔE) from the modified colorimetric measure was conducted using a linear mixed model (Equation 3) to test for significance of entry mean effects across environments that varied across year and time of sampling (low vs. high air temperatures). Because the data were not normally distributed, the color change (ΔE) was analyzed using the log transformation.

(3) $$Y_{\text{ijklmn}}=\mu +{\text{pedigree}}_{i}+{\text{year}}_{j}+{\text{replication}}_{k}+{\text{range}}_{l}+{\mathrm{row}}_{m}+{\text{batch}}_{n}+\text{pedigree}\;x\;{\text{year}}_{\mathrm{ij}}+\text{pedigree}\;x\;{\text{batch}}_{\mathrm{nj}}+{\text{error}}_{\text{ijklmn}}\;$$

Y represented individual observations of each pedigree, μ was the grand mean, and ${\text{pedigree}}_{i}$ represented effect of the pedigree (fixed). All other effects were random; ${\text{year}}_{j}$, effect of the year; ${\text{replication}}_{k}$, effect of the replication; ${\text{range}}_{l}$, effect of range; $\;{\mathrm{row}}_{m}$, effect of row; ${\text{batch}}_{m}$, effect of batch of HCN laboratory processing; $\text{pedigree}\;x\;{\text{year}}_{\mathrm{ij}}$ interaction, $\text{pedigree}\;x\;{\text{batch}}_{\mathrm{nj}}$ interaction; and ${\text{error}}_{\text{ijklmn}}$ represented the pooled error for all the mentioned factors in Equation (3).

The model was reduced by eliminating effects that did not minimize (<0.1%) error variation and non‐significant interaction effects (Equation 4).

(4) $$Y_{\text{ijklmn}}=\mu +{\text{pedigree}}_{i}+{\text{year}}_{j}+{\text{replication}}_{k}+{\text{range}}_{l}+{\text{batch}}_{n}+\text{pedigree}\;x\;{\text{year}}_{\mathrm{ij}}+{\text{error}}_{\text{ijklmn}}$$

A mean separation using a Student's *t*‐test was performed across the pedigrees to delimit pedigrees based on HCN concentrations. To compare HCN‐P produced across vegetative, flowering, and physiological maturity within one growth season, analysis within the year (2020) was performed as that was the only year where data could be taken at all three growth stages.

## 3 RESULTS AND DISCUSSION

### 3.1 Hydrogen cyanide colorimetric curve

**FIGURE 2:**
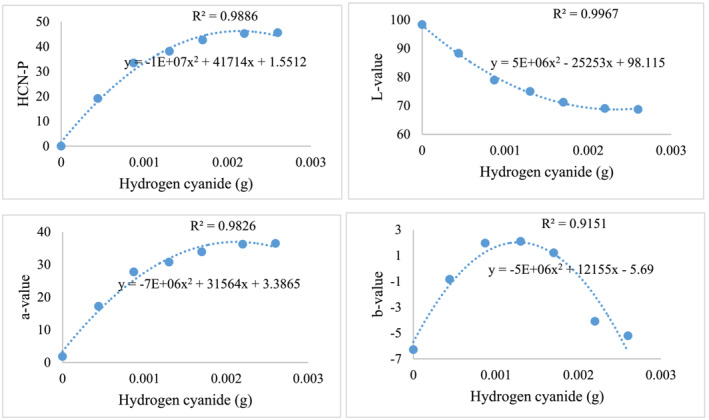
Relationship between HCN‐P (top‐left), L‐value (top‐right), a‐value (bottom‐left), and b‐value (bottom‐right) across the actual hydrogen cyanide from amygdalin to develop a predictive curve based on known standards. HCN‐P is calculated from the differences between a‐value, b‐values, and L‐values between post‐ and pre‐incubation images according to Equation (1).

The HCN colorimetric curve considered change in color (ΔE) by the action of β‐glucosidase enzyme versus the actual HCN from varying amygdalin concentrations (Karsavuran et al., 2014). An increase in HCN increased the a‐value (red‐green vector); the b‐value (blue‐yellow vector) increased up to HCN concentrations of 0.015 g at which point b‐values decreased with higher HCN concentration. Conversely, the L‐value decreased as HCN concentrations increased. The trends in colorimetry were consistent as reflected by high R^2^ values for all variables (Figure 2).

### 3.2 Comparisons between the visual color assessment assay and colorimetric measure

**FIGURE 3:**
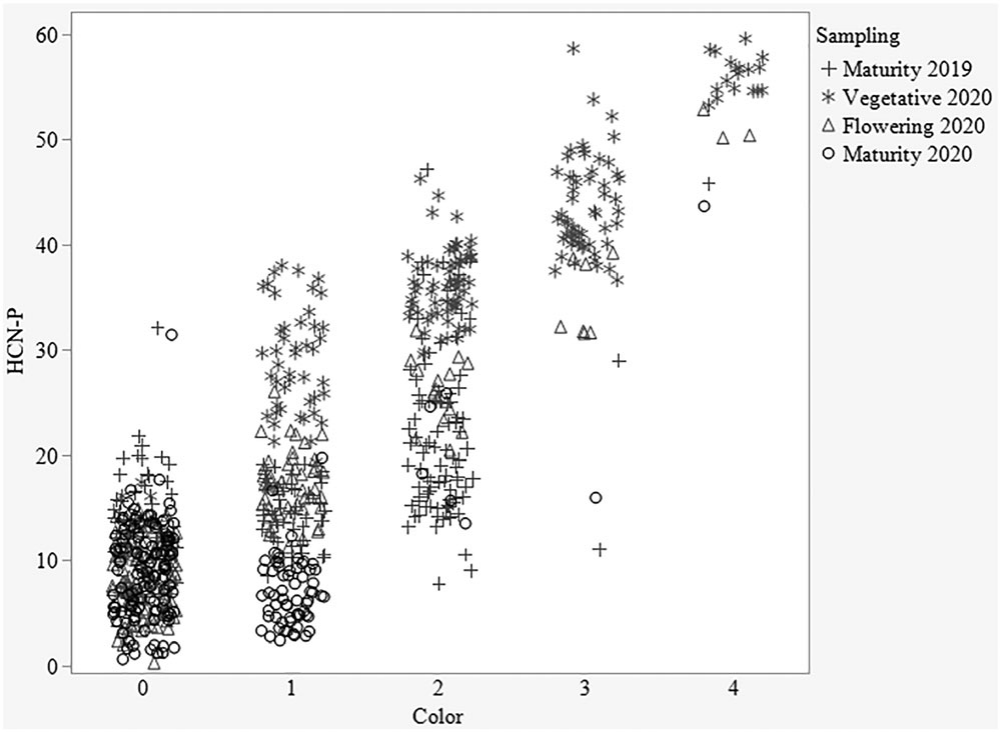
Colorimetric versus visual assessment assay across the growth stages for 2019 and 2020

The visual assessment and colorimetric measure assays were highly correlated (R^2^ = 0.83) across all sampling dates and thus produced similar results; for example, HCN‐P was lower in more mature sorghum. However, the visual color score consistently produced lower HCN‐P estimates than the colorimetric assay with a greater range in variation (Figure 3). The colorimetric measure was also found to have lower error variation than visual assessment (Table 3), improving the explained variation in the qualitative assay by 3%.

**TABLE 3:** Interpreting the color change in the visual assessment assay determining HCN concentration in the sorghum lines

Color scale	Indicator paper color change		
	Indicator paper	Interpretation	Inference
0	No color change	No detectable HCN	Safe for grazing
1	Very light golden‐brown	Very low HCN	Safe for grazing
2	Light brown/copper	Low HCN	Safe for grazing
3	Dark brown color	Moderate HCN	Should be grazed sparingly, not safe if allowed in large quantities
4	Very dark brown/burgundy	Very high HCN	Not safe for grazing

Bradbury et al. (1999) documented different methods of HCN estimation ranging from picrate paper tests to spectrometry and acid hydrolysis. In comparison with the methods used in this study, the picrate paper tests used in Bradbury et al. (1999) had longer exposure times ranging from 3 (shortest) to 24 h, which is prohibitively time consuming when dealing with large numbers of genotypes. A similar approach was also used by Burns et al. (2012) with the same challenge. Spectrometry was used at a later stage to quantify the color change of the picrate paper by preparing aqueous solutions to record absorbance, and this improved results though is also more labor intensive. This approach requires more than a day to get results, which can become resource intensive in the long run (Bradbury et al., 1999; Burns et al., 2012; Fox et al., 2012). Further, the authors (Bradbury et al., 1999) found that the potassium phosphate used in the acid hydrolysis approach reduced the rate of liberation of HCN from the cyanogenic glucoside of the cassava flour and reduced accuracy of the assay.

Methods used in this study (colorimetry and visual assessment) were faster than the previous studies, because results obtained within an hour of laboratory work, freeing up space, glassware, and time to increase the number of samples. Colorimetry only added approximately 2 min per sample. It has been found that lower accuracy methods can still result in better decisions if offset by higher throughput and low cost that allows greater sample collection (Lane & Murray, 2021).

In addition, the colorimetric assay provided both improved accuracy and quantitative metrics important for statistical analyses and for selection in a breeding nursery. Most importantly, visual assessment is a subjective rating subject to variation from one evaluator to another; consequently, results are often less reproducible (Johnston & Kao, 1989).

### 3.3 Heritability/repeatability of HCN

In analysis, none of the main effects or interactions were significant for visual assessment but significant effects were detected for year, pedigree and pedigree × year interaction in the colorimetric analysis (Table 3). Given the absence of genetic variation in the visual assay, the repeatability was zero; in the colorimetric assay, repeatability for HCN‐P, although present, remained low. It is hard to estimate genetic variation in a qualitative assay (visual assessment) because it is categorical in nature, and it was not surprising that the repeatability was zero. However, the low repeatability of the colorimetric assay is largely explained by the large differences between the years, pedigrees, and growth stages and interactions between these differences. As mentioned previously, environmental stress, which differs from year to year, as well as the phenological stage the plant is in will alter the HCN‐P. Repeatability estimates could be improved by only comparing sampling points within a similar growth stage. However, manipulating sampling or analysis solely to improve repeatability would be ill advised. From an agronomic perspective, especially under grazing systems where the crop might be consumed at any time, it is important that the pedigrees have low HCN across all growth stages. In the case of this study, however, HCN‐P estimation in 2019 was only done at maturity. If sampling had been done at five‐ to seven‐leaf stage and flowering as in 2020, this could have increased reproducibility (Table 4).

**TABLE 4:** Comparison between the visual assessment assay and colorimetric measure for % variation, significance, and repeatability across 2019 and 2020

	Visual color scale		Colorimetric (HCN‐P)	
Source of variation	% variation	Significance	% variation	Significance
Year	28.4	0.4853	28.8	<0.001
Pedigree	0.0	0.0865	1.5	0.0009
Pedigree × year	4.1	0.3173	25.3	0.0005
Batch	11.4	0.0639	0	0.7498
Replication	1.5	0.7754	0.9	0.597
Range	11.4	0.0989	2.2	0.4797
Row	2.8	0.0857	0	0.4637
Pedigree × batch	10.1	0.3027	14.2	0.4809
Error	30.2		27.1	
Repeatability	0.0		.16	

### 3.4 Comparison between years

**FIGURE 4:**
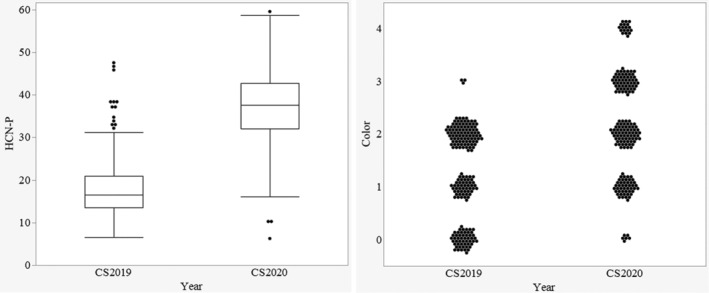
Comparison between the HCN‐P distribution for the colorimetric measure (left) and the color scores of the visual assessment across plots for 2019 (high air temperatures) and 2020 (lower air temperatures) (right)

The raw data for HCN‐P ranged from 7 to 47 for 2019 (September 3–6) and from 6 to 60 for 2020 (May 27–29) for the colorimetric measure (Figure 4). Average HCN‐P from 2019 (16) was significantly lower than in 2020 (31). The year and pedigree × year effect accounted for 29% and 25%, respectively, of the total experimental variation (Table 3). Overall, HCN‐P increased in 2020. This difference was clearly shown by the frequencies of plots scoring in the various color categories of the visual assessment, which showed most of the plots had no to very low HCN‐P in 2019 and a significantly higher HCN‐P across all color categories in 2020 (Figure 4). Pedigrees performed significantly differently from one another for the colorimetric HCN‐P, meaning there is variation and selection can be done for low HCN perennial sorghums.

**FIGURE 5:**
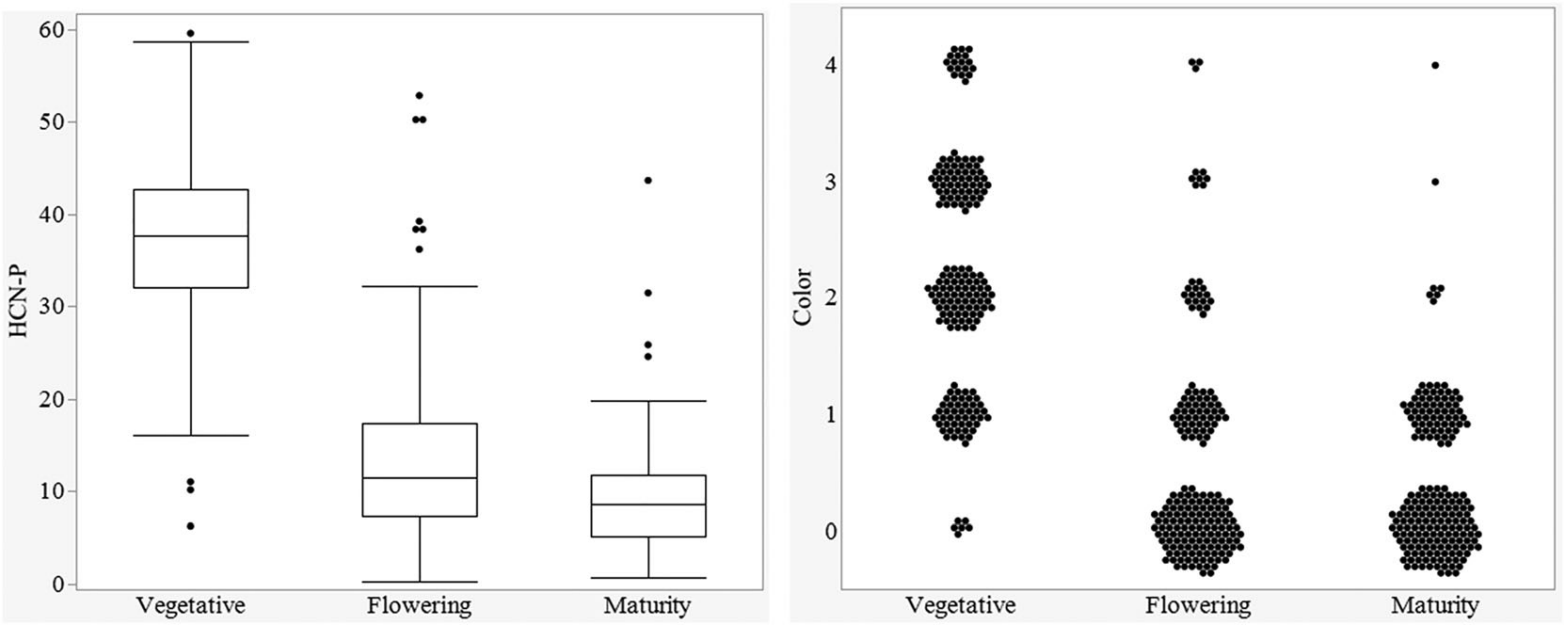
Comparison between the HCN‐P distribution across growth stages of the colorimetric measure (left) and the color scores of the visual assessment across plots (right) in 2020

This difference between years might be explained in part by the difference in air temperatures during sampling between late summer of the first season and late spring sampling of the second season, in addition to growth stage differences. Zahid et al. (2012) showed higher HCN‐P in the earlier plant growth stages and HCN concentrations decreased as biomass increased in later growth phases. Within 2020, the growth stage significantly affected HCN‐P, accounting for 72% of the total experimental variation. The highest HCN‐P was recorded in the vegetative phase, with concomitant decreases in both flowering and maturity stages (Figure 5). HCN‐P of the individual pedigrees was consistent across the growth stages per se (*P* = 0.5). The comparison made across the growth stages confirmed the findings of Zahid et al. (2012), as the HCN‐P reduced with continued growth from vegetative to physiological maturity.

### 3.5 Comparisons between perennial and annual sorghums

Among sorghum pedigrees, several trends became obvious. First, most of the grain sorghum pedigrees were low in HCN‐P (Table S1). The lower HCN‐P in grain sorghums may be due to selective breeding; breeding for adaptation could have indirectly selected against dhurrin content. These results are similar to Hayes et al. (2016) who concluded that wild sorghums have a higher HCN‐P when stressed. Given this perspective, intentional phenotyping, selection, and breeding for low HCN perennial sorghum will be necessary to produce genotypes acceptable to animal producers.

## 4 CONCLUSION

Colorimetry is an objective and more precise method to assess the HCN‐P of sorghum forages compared with visual color assessment. Overall, HCN‐P had a low repeatability across seasons, implying that additional refinement of the technique is merited, but this problem might be offset by the high throughput and low cost of the chemical methods used (Lane & Murray, 2021). Both annual and perennial sorghum produce dhurrin, but perennial sorghum had significantly higher HCN‐P than annual sorghum. If the trait is heritable, selection for pedigrees with reduced HCN‐P is important.

Other factors also influence HCN‐P. First, environmental conditions throughout the year affect HCN‐P in sorghum, but pedigree × year interaction indicated that pedigrees reacted differently to these conditions. Growth stage maybe even more important and is important to consider when feeding animals on fresh forage. Perennial sorghum should either be processed into silage or hay or given sparingly in the earlier growth cycle.

## CONFLICT OF INTEREST

The authors declare no conflicts of interest regarding publication of this manuscript.

## Supporting information


**Table S1:** Best Linear Unbiased Estimates for the quantified color change (E) produced for perennial and grain sorghum across 2019 and 2020.
