# Supplementary material for: Genetic variation in hydrogen cyanide potential of perennial sorghum evaluated by colorimetry
Source: Plant Direct. 2022 Oct 21;6(10):e448. doi: 10.1002/pld3.448 (PMC9587379; doi:10.1002/pld3.448)
Supplement: Supplementary file 1 — Table S1: Best Linear Unbiased Estimates for the quantified color change (E) produced for perennial and grain sorghum across 2019 and 2020. [file PLD3-6-e448-s001.pdf]

Supplemental Table 1: Best Linear Unbiased Estimates for the quantified color change (E) produced for perennial and grain sorghum across 2019 and 2020.

	PEDIGREE	ΔE	SE		PEDIGREE	ΔE	SE
1	S3317-B2-PR65A	38.84	0.19	51	S2372-5-R651	24.86	0.19
2	S1662>R126B	34.94	0.19	52	S3322-C6-PR67	24.83	0.19
3	S14PR-020C	34.90	0.19	53	S2002>269D	24.36	0.19
4	S1662>R153G	32.19	0.19	54	S2172-7-R475DW	24.30	0.19
5	S1662>R040A-R161	32.14	0.19	55	S1776>349C	24.19	0.19
6	CS14-PSOR-080-15	31.88	0.19	56	S1661>089	24.15	0.19
7	S2172-5-R506	31.58	0.19	57	S1327>159A	24.14	0.19
8	S1820-1-614	31.44	0.19	58	RTx437	23.71	0.23
9	S2371>R519-R124	30.92	0.19	59	S1843-8-578C	23.60	0.19
10	CS14-PSOR-022-15	30.88	0.19	60	S2607-1-R64A	23.40	0.19
11	X1083-021	30.53	0.19	61	S1346>174A	23.29	0.19
12	S1662>246A	30.43	0.19	62	S1477>R216	23.27	0.19
13	X814>R209	30.23	0.19	63	X1210-354	23.23	0.19
14	X814-201B-PR9C	29.61	0.19	64	X799>105	23.17	0.19
15	S1479>R771A	29.61	0.19	65	S1341>R315	23.11	0.19
16	X1092-154	29.55	0.19	66	S2171-23-R545A	22.79	0.19
17	S3188-B6-PR46A	29.21	0.19	67	S1852>015B-R171A	22.79	0.19
18	S2069-3>BK126	29.11	0.19	68	S2371-23-R545C	22.77	0.19
19	S1479>R334B-R45	28.98	0.19	69	S1662>R246C	22.41	0.19
20	S2092-1-115BK-R86	28.96	0.19	70	S1776>R65	22.35	0.19
21	X999>R393	28.93	0.19	71	S3199-B1-PR135A	22.34	0.19
22	S2172>R187	28.65	0.19	72	S2125>245B	22.33	0.19
23	S3182-B7-PR178A	28.65	0.19	73	S1662>153H-R54	22.25	0.19
24	S1327>230B	28.11	0.19	74	S3195-A3-PR14	22.07	0.19
25	X814>R003	27.91	0.19	75	S2126>R147B	22.00	0.19
26	S3167-B1-PR10G	27.90	0.19	76	S1814>R452B	21.99	0.19
27	S2163-1-065-R190	27.64	0.19	77	S1852>015E	21.92	0.19
28	S1327>R144	27.47	0.19	78	S2372-4-R650A	21.84	0.19
29	S1662>R512A-R98	27.30	0.19	79	S1477>R175A	21.44	0.19
30	X1206-054	27.25	0.19	80	X999>R305	21.41	0.19
31	X38-18-R4-193	27.19	0.19	81	X814-201C-R17	20.85	0.19
32	S1438>070A	27.04	0.19	82	X814-201B-R523	20.75	0.19
33	X999>177B	26.95	0.19	83	S1465>R120D	19.99	0.19
34	S2172-5>R312	26.91	0.19	84	S1662>216	19.53	0.19
35	S1438>R302	26.79	0.19	85	S2097-4-137	19.20	0.19
36	S1479>R334B	26.77	0.19	86	S14PR>R181	18.64	0.19
37	S1312>R97A	26.39	0.19	87	X999R348B	17.25	0.19
38	S1465>587A-PR125A	26.14	0.19	88	S1383>046F	16.71	0.19

39	S1776>R349B	26.11	0.19	89	S2163>R186B-R129G	15.14	0.19
40	X1206>R477	26.06	0.19	90	CS14-PSOR-091-03	13.22	0.19
41	S1776-R331	25.99	0.19	91	RTx436	12.90	0.23
42	S1852>015B-R171B	25.91	0.19	92	S1776>R174*	12.70	0.19
43	S1477>605A	25.62	0.19	93	GYPSUM-9	11.77	0.19
44	S1344>R036	25.53	0.19	94	NP32-P15355776	11.22	0.19
45	X754-198	25.46	0.19	95	X999>R485	10.98	0.19
46	S2371-81-R606A	25.36	0.19	96	S1662>R554B	9.15	0.19
47	S3182-B3-PR39B	25.31	0.19	97	X814-201A-PR101C	6.02	0.19
48	S2371-98-R623	25.30	0.19	98	BTX623(4X)	3.52	0.19
49	S1662>R040A	25.14	0.19	99	LowHCN	3.14	0.19
50	S1646>R46	25.02	0.19	100	BTX623	2.77	0.19

* SE-Standard error, highlighted in red are the 5% highest hydrogen cyanide producing pedigrees and those in yellow are the grain sorghums
